# Reviewer List for 2025

**DOI:** 10.1111/vec.70098

**Published:** 2026-02-10

On behalf of the countless individuals tasked with making the *Journal of Veterinary Emergency and Critical Care* a success, we wish to sincerely thank all of our reviewers who contributed to the success of the journal in 2025. Your contributions to the peer review process are invaluable in our pursuit of excellence. We would like to especially thank those reviewers who reviewed five or more manuscripts this year—your dedication to the journal is commendable. The number of reviews performed is noted in parentheses.

Mays, Erin (12)

Cook, Simon (10)

Donati, Pablo (10)

Walton, Rebecca L. (10)

Hezzell, Melanie Jane (9)

Humm, Karen (9)

Nash, Katherine (9)

Rozanski, Elizabeth (9)

Her, Jiwoong (8)

Monnig, Andrea (8)

Blois, Shauna (7)

Hopper, Kate (7)

Jandrey, Karl (7)

Jones, Nadine (7)

Marshall, Hannah (7)

Turley, Kelsey A. (7)

Bishop, Rebecca (6)

Brainard, Benjamin (6)

Cuddy, Lindsay (6)

Epstein, Steven (6)

Keir, Iain (6)

Klainbart, Sigal (6)

Lynch, Alex M. (6)

O'Kelley, Benjamin (6)

Pfaff, Alexandra (6)

Wismer, Tina (6)

Zeiler, Gareth E. (6)

Aumann, Samantha (5)

Bracker, Kiko (5)

Chan, Dan (5)

Dicker, Samuel Avram (5)

Hovda, Lynn (5)

Jagodich, Tiffany (5)

Lyons, Bridget (5)

Pigott, Armi (5)

Reinhart, Jennifer M. (5)

Stastny, Tereza (5)

Wardrop, K. Jane (5)

Zersen, Kristin (5)

Boag, Alisdair (4)

Boag, Amanda (4)

Bowen, Caitlin (4)

Briganti, Angela (4)

Conner, Bobbi (4)

Dörfelt, René (4)

Downing, Frances (4)

Dupanloup, Adrien (4)

Fischer, Andrea (4)

Fletcher, Dan (4)

Guillaumin, Julien (4)

Halpin, Rachel (4)

Hasegawa, Daisuke (4)

Hemmelgarn, Carey Ann (4)

Hoehne, Sabrina (4)

Hurn, Simon (4)

Jones, Jessica (4)

Judy, Annalisa (4)

Kaplan, Amy (4)

Kelley, Morgan (4)

Knipe, Marguerite F. (4)

Levy, Nyssa (4)

Molitoris, Amy (4)

Munsterman, Amelia (4)

Odunayo, Adesola (4)

Oricco, Stefano (4)

Partlow, Jessica (4)

Quandt, Jane (4)

Safrany, Ben (4)

Sigrist, Nadja (4)

Turner, Kate (4)

Ueda, Yu (4)

Wheeler, Lance R. (4)

Wilkins, Pamela A. (4)

Wright, Ian (4)

Zoff, Aurora (4)

Alwood, Ames (3)

Asokan, Vibha (3)

Bae, Seulgi (3)

Bateman, Shane (3)

Bates, Nicola (3)

Beal, Matthew (3)

Bell, Amy (3)

Birkbeck, Rachael (3)

Blong, April (3)

Boller, Manuel (3)

Bucknoff, Melissa (3)

Burchell, Richard (3)

Camacho, Fernanda (3)

Cazzolli, Dava (3)

Daly, Meredith (3)

Davidow, Elizabeth (3)

Destefano, Ian M. (3)

Farrell, Kate (3)

Forbes, Jethro (3)

García San José, Paula (4)

Goodnight, Michelle (3)

Julie, Menard (3)

Lee, Justine (3)

McLaughlin, Christopher Matthew (3)

McMichael, Maureen (3)

Ngwenyama, Thandeka (3)

Ozawa, Sarah (3)

Parkinson, Lily (3)

Philp, Helen Suzette (3)

Schaer, Michael (3)

Schoeman, Johan (3)

Sharp, Claire (3)

Talbot, Charles (3)

Tello, Luis (3)

Waddell, Lori (3)

Wayne, Annie (3)

Woolcock, Andrew (3)

Yozova, Ivayla (3)

Adin, Darcy (2)

Anastasio, John (2)

Anderson, Kimberley (2)

Becker, Jami (2)

Boyd, Corrin (2)

Boysen, Søren (2)

Brockman, Daniel (2)

Burgess, Brandy (2)

Casale, Sue (2)

Cooper, Edward (2)

Cortellini, Stefano (2)

Daly, Jennifer (2)

Davros, Kat (2)

Dickerson, Vanna (2)

Doss, Grayson A. (2)

Edwards, Thomas H. (2)

Ellis, Connor (2)

Fink, Lisa (2)

Frauenthal, Virginia Marie (2)

Gant, Poppy (2)

Gleeson, Molly (2)

Hagley, Simon (2)

Hansen, Bernard (2)

Hansen, Sonya (2)

Hanson, Reid (2)

Hennelly, Emiliana (2)

Hickey, Mara (2)

Ilie, Laura (2)

Johnson, Justine (2)

Johnson, Lynelle (2)

Kazakos, George (2)

Kerl, Marie (2)

Koid, Audrey (2)

Loewen, Jennifer (2)

Long, Alicia (2)

Love, Lydia C. (2)

Lucchetti, Brittany (2)

Murphy, Lisa (2)

Olin, Shelly (2)

Peck, Courtney M. (2)

Prieto, Jennifer M. (2)

Rechy, Jaime (2)

Respess, Meghan (2)

Rosser, Mike (2)

Rush, John E. (2)

Rutter, Christine (2)

Schaefer, Jonathan (2)

Silverstein, Deb (2)

Stanley, Monique (2)

Stanzani, Giacomo (2)

Starybrat, Daria (2)

Thomas, Emily (2)

Toribio, Ramiro (2)

Wallace, Mandy (2)

Walton, Jenny (2)

Weese, Scott (2)

Weltman, Joel (2)

Whitney, Joanna (2)

Winter, Randolph (2)

Woliver, Cory (2)

Wong, Eileen (2)

Yoon, Hajeong (2)

Acierno, Mark (1)

Adamik, Katja (1)

Adamovich‐Rippe, Krista Nicole (1)

Alwood, Amy (1)

Ames, Marisa (1)

Attipa, Charalampos (1)

Baker, J. A. (1)

Balakrishnan, Anusha (1)

Barbosa, Vivian Fernanda (1)

Bernhard, Christa (1)

Black, Dorothy (1)

Blutinger, Alex (1)

Bordin, Angela (1)

Bruno, Barbara (1)

Burke, Jasper (1)

Celliers, Anri (1)

Chalifoux, Nolan V. (1)

Chow, Rosalind (1)

Clarke, Carolyn (1)

Clarkin‐Breslin, Rachel C. (1)

Constable, Peter D. (1)

Crabtree, Naomi E. (1)

D., Spector (1)

Davis, Megan (1)

Dechant, Julie (1)

Delvescovo, Barbara (1)

Deschamps, Jack‐Yves (1)

Dias Moreira, Ana Sofia (2)

Donaldson, Rebekah (1)

Doulidis, Pavlos G. (1)

Ellerbrock, Robyn (1)

Estevam, Marina (1)

Fielding, C. Langdon (1)

Fischetti, Anthony (1)

Flores, Rebecca (1)

Foster, J. D. (1)

Geddes, Rebecca (1)

Gerken, Katherine (1)

Gladden, Jay (1)

Gomes Lourenço, Maria Lucia (1)

Granfone, Marcella (1)

Griebsch, Christine (1)

Hall, Kelly E. (1)

Harmon, Meghan (1)

Higgs, Veronica Ann (1)

Humphrey, Sarah (1)

Jensen, Asger Lundorff (1)

Kelmer, Efrat (1)

Labato, Mary Anna (1)

Landry, Greg M. (1)

Lane, Selena L. (1)

Larson, Melinda J. (1)

Linklater, Andrew (1)

Mandell, Debbie (1)

Mann, F. Tony (1)

Marks, Stanley (1)

Mazzaccari, Kaitlyn (1)

McCobb, Emily (1)

McKenzie, Erica C. (1)

Merbl, Yael (1)

Murphy, Kellyann (1)

Osekavage, Katie E. (1)

Passantino, Annamaria (1)

Paul, April (1)

Perry, Brittany (1)

Petritz, Olivia (1)

Prisk, Amanda Jane (1)

Ralphs, S. Christopher (1)

Sanclemente, Jorge Luis (1)

Schmid, Renee (1)

Schott, Harold (1)

Scott, Victoria (1)

Seo, Joonbum (Joon) (1)

Shaw, Scott (1)

Spalla, Ilaria (1)

Tan, Avalene (1)

Tart, Kelly (1)

Thomer, Amanda (1)

Thomovsky, Elizabeth J. (1)

Tocci, Lynel (1)

Tonozzi, Caroline (1)

Vezzi, Noel (1)

Walker, Julie Michele (1)

Wallace, Mary (1)

Webb, Tracy L. (1)

Wegenast, Colette A. (1)

Wells, Raegan (1)

Whelan, Megan (1)

Wolf, Jacob (1)

Yang, Kitty (1)

Yaxley, Page E. (1)

